# Exploring pathological signatures for predicting the recurrence of early-stage hepatocellular carcinoma based on deep learning

**DOI:** 10.3389/fonc.2022.968202

**Published:** 2022-08-19

**Authors:** Wei-Feng Qu, Meng-Xin Tian, Jing-Tao Qiu, Yu-Cheng Guo, Chen-Yang Tao, Wei-Ren Liu, Zheng Tang, Kun Qian, Zhi-Xun Wang, Xiao-Yu Li, Wei-An Hu, Jian Zhou, Jia Fan, Hao Zou, Ying-Yong Hou, Ying-Hong Shi

**Affiliations:** ^1^ Department of Liver Surgery, Liver Cancer Institute, Zhongshan Hospital, Fudan University, Key Laboratory of Carcinogenesis and Cancer Invasion of Ministry of Education, Shanghai, China; ^2^ Department of General Surgery, Zhongshan Hospital, Fudan University, Shanghai, China; ^3^ Tsimage Medical Technology, Yihai Center, Shenzhen, China; ^4^ Department of Information and Intelligence Development, Zhongshan Hospital, Fudan University, Shanghai, China; ^5^ Center for Intelligent Medical Imaging & Health, Research Institute of Tsinghua University in Shenzhen, Shenzhen, China; ^6^ Department of Pathology, Zhongshan Hospital, Fudan University, Shanghai, China

**Keywords:** hepatocellular carcinoma, curative resection, recurrence, deep learning, pathological slides

## Abstract

**Background:**

Postoperative recurrence impedes the curability of early-stage hepatocellular carcinoma (E-HCC). We aimed to establish a novel recurrence-related pathological prognosticator with artificial intelligence, and investigate the relationship between pathological features and the local immunological microenvironment.

**Methods:**

A total of 576 whole-slide images (WSIs) were collected from 547 patients with E-HCC in the Zhongshan cohort, which was randomly divided into a training cohort and a validation cohort. The external validation cohort comprised 147 Tumor Node Metastasis (TNM) stage I patients from The Cancer Genome Atlas (TCGA) database. Six types of HCC tissues were identified by a weakly supervised convolutional neural network. A recurrence-related histological score (HS) was constructed and validated. The correlation between immune microenvironment and HS was evaluated through extensive immunohistochemical data.

**Results:**

The overall classification accuracy of HCC tissues was 94.17%. The C-indexes of HS in the training, validation and TCGA cohorts were 0.804, 0.739 and 0.708, respectively. Multivariate analysis showed that the HS (HR= 4.05, 95% CI: 3.40-4.84) was an independent predictor for recurrence-free survival. Patients in HS high-risk group had elevated preoperative alpha-fetoprotein levels, poorer tumor differentiation and a higher proportion of microvascular invasion. The immunohistochemistry data linked the HS to local immune cell infiltration. HS was positively correlated with the expression level of peritumoral CD14^+^ cells (*p*= 0.013), and negatively with the intratumoral CD8^+^ cells (*p*< 0.001).

**Conclusions:**

The study established a novel histological score that predicted short-term and long-term recurrence for E-HCCs using deep learning, which could facilitate clinical decision making in recurrence prediction and management.

## Introduction

Hepatocellular carcinoma (HCC) is the sixth most common malignancy and the fourth leading cause of cancer related deaths worldwide (1). Several treatments including surgery, locoregional therapies, and immunotherapy have been adopted as standards of care according to different tumor stages (2–4). Curative resection offers a chance of improved survival for HCC patients, especially those with early-stage tumors that are defined as Barcelona Clinic Liver Cancer (BCLC) stages 0 and A (5). Although the 5-year survival rate can reach 70% in early-stage HCC patients (6), half of patients suffer recurrence after liver resection (7) due to the lack of approved adjuvant therapies. Therefore, the precise prediction of postoperative recurrence is urgently needed.

Common prognostic factors for predicting early-stage HCC recurrence include pathological features, clinical biomarkers and genetic signatures (8). Recently, Yuan et al. developed a CpG methylation signature to elucidate the recurrence patterns in early-stage HCC with concordance indexes (C-indexes) of approximately 0.7 in three datasets (9). Compared to traditional staging systems, radiomics models also showed favorable efficacy in the recurrence prediction of HCC patients within the Milan criteria (10). In terms of histological features, specific structures such as microvascular invasion (MVI) and tumor-infiltrating lymphocytes were associated with recurrence risk (11, 12). However, due to the high heterogeneity in HCC, valuable information based on whole-slide images (WSIs) has not been thoroughly detected. Furthermore, the correlation between pathological texture and recurrence remains unknown.

In the past decade, breakthroughs in artificial intelligence (AI) have made remarkable progress in cancer research (13, 14). With the increasingly high capacity of deep learning, a large amount of work involved in the histopathological fields has been carried out, including tumor diagnosis, subtyping, grading, staging, and prognostic prediction (15–17), as well as the identification of pathological features, biomarkers, and genetic changes (18, 19). The advent of digital WSIs of tissue has not only economized the great amount of time or manual labor needed but also potentiated mining of subvisual morphometric phenotypes and ultimately improved patient management or therapeutic decision-making. Previous studies have originally proposed survival indicators based on digital WSIs *via* deep learning (20, 21). Nevertheless, the existing computational methods can identify only basic HCC structures. Complex and rich information contained in architectural features such as the portal area and lymphocytes is still hampered to be expounded. Herein, we explored more distinctive histological features to further describe the recurrence patterns and clinicopathological information of early-stage HCC.

The present study successfully developed a convolutional neural network (CNN) based on six classes of HCC tissues (namely, tumor region, normal liver tissue, portal area, fibrosis, hemorrhage/necrotic area, and lymphocyte area) and constructed a histological score (HS) *via* least absolute shrinkage and selection operator (LASSO) Cox regression to assess patients’ recurrence risk after hepatectomy. The novel model was validated in the three independent cohorts. By stratifying patients into different risk subgroups, their prognosis could be precisely appraised and multiomics characteristics were investigated.

## Materials and methods

### Patient cohort and study design

**Figure 1:**
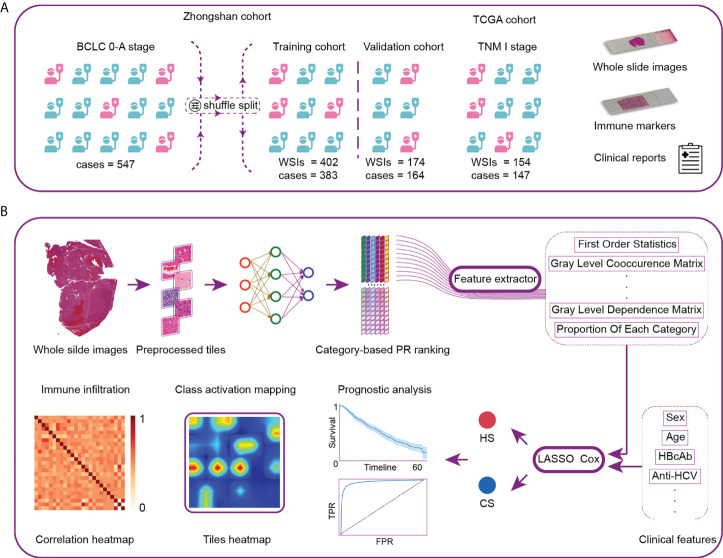
Workflow and general methodology of the study. **(A)** The recurrence-related scores were first developed and internally validated in a series of patients with BCLC stage 0-A treated by curative resection at Zhongshan Hospital. The scores were then externally validated by TNM-I stage patients in the TCGA cohort. **(B)** We first developed the neural network using 116 whole-slide images (WSIs) as the category-based training data. The network was then used to analyze the remaining WSIs and generate the classification maps. Pathological image features were extracted from typical tiles. Next, we constructed two recurrence prediction scores *via* LASSO-Cox. CS was composed of clinical and histological characteristics. HS was developed based on pure histological features. Finally, we analyzed the model discrimination ability, patient prognosis and local immune cell infiltration. TCGA, The Cancer Genome Atlas; PR, precision-recall; CS, combined score; HS, histological score; TPR, true positive rate; FPR, false positive rate.

A total of 416 WSIs and 387 corresponding early-stage HCC patients who underwent radical resection at Zhongshan Hospital from January 2006 to December 2011 were retrospectively enrolled as the first dataset (**Figure 1**). The inclusion criteria were as follows: 1) pathologically proven HCC; 2) no neoadjuvant antitumor therapy; 3) Child–Pugh class A or B before surgery; and 4) BCLC stage 0-A. The exclusion criteria were as follows: 1) presence of other pathological types, such as intrahepatic cholangiocarcinoma (ICC) or combined hepatocellular cholangiocarcinoma (CHC); 2) previous antitumor treatment; 3) missing clinical information; and 4) death or disease recurrence within 1 month after resection. Data on tumor stages were collected according to the BCLC staging system (22) and China Liver Cancer (CNLC) (23) staging guidelines.

Following the same inclusion and exclusion criteria, another 160 patients who underwent curative partial hepatectomy at Zhongshan Hospital from October 2014 to December 2014 and 160 WSIs were enrolled as the second dataset. We combined the two datasets and randomized cases into the training and validation cohorts at a ratio of 7:3.

We enrolled 154 WSIs and 147 patients from The Cancer Genome Atlas (TCGA) database as the external validation cohort (https://portal.gdc.cancer.gov/). Due to the lack of information on BCLC staging, all the enrolled cases had tumor node metastasis (TNM) stage I disease, which fulfilled the criterion of BCLC stage 0-A.

The follow-up was censored in December 2019. Recurrence was the primary endpoint in the present study. HCC recurrence was defined as the appearance of a newly detected HCC tumor confirmed on two radiologic images, with or without an elevation in serum tumor markers. Time to recurrence (TTR) was defined as the time between surgery and recurrence or metastasis. Recurrence-free survival (RFS) and overall survival (OS) were the secondary endpoints. RFS was defined as the time from the date of hepatectomy to the date of recurrence, metastasis, death, or the last follow-up. OS was defined as the time between resection and death. The study obtained ethical approval from the Institutional Review Board of Zhongshan Hospital and complied with the standards of the Declaration of Helsinki. Informed consent was received from each patient before surgery.

### Preparation of H&E staining and immunohistochemistry for immune markers

Hematoxylin and eosin (H&E) staining was performed on paraffin-embedded tissues that were at 4 µm thickness. Tissue microarray (TMA) construction and immunohistochemistry for 28 immune markers were conducted as previously described (11, 24). Some of the previous data were used directly as a complement to the results (11).

### Image annotation and processing

We randomly selected 116 WSIs for annotation in the Zhongshan cohort. Using ASAP 1.8, two pathologists manually annotated and fully examined the slides in six categories: tumor region, normal liver tissue, portal area, fibrosis, hemorrhage/necrotic area, and lymphocyte area. The annotated WSIs were divided into training, validation, and testing datasets at a ratio of 8:1:1. The annotated tissue areas were extracted based on the binary mask obtained by OTSU (25) and then divided into small squares, 299 pixels×299 pixels in size, called “tiles”. After image incision, data enhancement methods were used to balance the number of tiles for the six categories (details are provided in the **Supplementary Methods**).

### Standardization of TCGA diagnostic slides

Due to the disparity in the staining and scanning process between the Zhongshan and TCGA cohorts, the trained neural model could not be directly applied to the TCGA WSIs. We modified the traditional Reinhard algorithm (26) to standardize stainingin both the Zhongshan and TCGA cohorts.

### Classification network

We mainly proposed training a classification network to discriminate six types of HCC tissues. Inception V3 (27) was used as the basic model. Classification maps were derived after image recognition. Morphological processing was utilized to optimize the original classification maps. We used the t-distributed stochastic neighbor embedding (t-SNE) algorithm to visualize the segmental results. The pathological signatures were then extracted from the ten tiles with the highest prediction probability for each type (details are provided in the **Supplementary Methods**).

### Establishment of the histological score and combined score

LASSO Cox regression (9) was applied to obtain high-dimensional prognostic features in histology. With recurrence status and TTR as labels, a combined score (CS) was constructed by integrally analyzing the histological signatures and clinical markers. The clinical markers included sex, age, serum levels of alpha-fetoprotein (AFP) and alanine transaminase (ALT), and morphological information such as tumor number and size. In the same way, we constructed a histological score (HS) through pure histological features (details are provided in the online **Supplementary Materials**). The optimal cutoff value for survival time was obtained through the “survminer” package. The patients were then divided into high-risk and low-risk subgroups for further survival comparisons.

### Correlation between HS and immune infiltration

To evaluate the correlation between the HS and immune infiltration conditions, we applied hierarchical clustering analysis for specific immune markers in the TMA. In the TCGA datasets, the CIBERSORT algorithm (28) was used to explore the quantity of tumor-infiltrating immune cells based on the transcriptome signature.

### Statistical analysis

Continuous variables are expressed as the median (IQR) and were compared with using Mann–Whitney U test. Categorical variables are expressed as numbers and percentages, and were compared with the χ^2^ test or Fisher’s exact test. Kaplan–Meier curves with the log-rank test were used to compare survival. The LASSO Cox method was used to select independent factors associated with recurrence. Hazard ratios (HRs) and 95% confidence intervals (CIs) were also estimated by means of univariable and multivariable Cox analyses. A two-tailed *p* value> 0.05 was considered statistically significant. Model discrimination was assessed by the overall C-index, receiver operating characteristic (ROC) curve and net reclassification improvement (NRI) (29). Statistical analysis was performed using R-software 3.6.3 (R Foundation, Vienna, Austria) and SPSS ^®^ 22.0 (IBM, Armonk, New York, USA).

## Results

### Patient demographics and clinical information

**Supplementary Table S1** describes the demographic, clinical, and tumor characteristics of patients in the training and validation cohorts. The median ages in the training and validation cohorts were 53 years and 55 years, respectively. Most patients in both cohorts were male. More than 80% of patients were infected with hepatitis B virus (HBV) and diagnosed with liver cirrhosis. Over half of the patients presented with elevated serum AFP levels in both cohorts (55.6% and 59.8%). Microvascular invasion was detected in 23.2% and 26.8% of patients in the training and validation cohorts, respectively. A majority of patients had a single lesion and were diagnosed as CNLC stage Ia. More patients were diagnosed with BCLC stage A than stage 0 in both cohorts. No significant differences were observed for the demographic information between the two cohorts.

After a median follow-up of 54.2 months (range, 3.0 to 64.6) for the Zhongshan population, 44.4% of patients (243/547) suffered tumor recurrence and 25.8% (141/547) died. The 1-, 3-, and 5-year recurrence rates were 17.1%, 33.1%, and 48.1%, respectively, and the 1-, 3-, and 5-year RFS rates were 82.2%, 66.4%, and 51.5%, respectively.

In terms of the TCGA cohort, the median follow-up time was 27.0 months (range, 1.1 to 115.9). A total of 38.8% of patients (57/147) suffered tumor recurrence and 21.1% (31/147) died. The 1-, 3-, and 5-year recurrence rates were 19.2%, 45.4%, and 62.0%, respectively, and the 1-, 3-, and 5-year RFS rates were 76.7%, 49.5%, and 34.5%, respectively.

### Visualization of category-based HCC tissue

**Figure 2:**
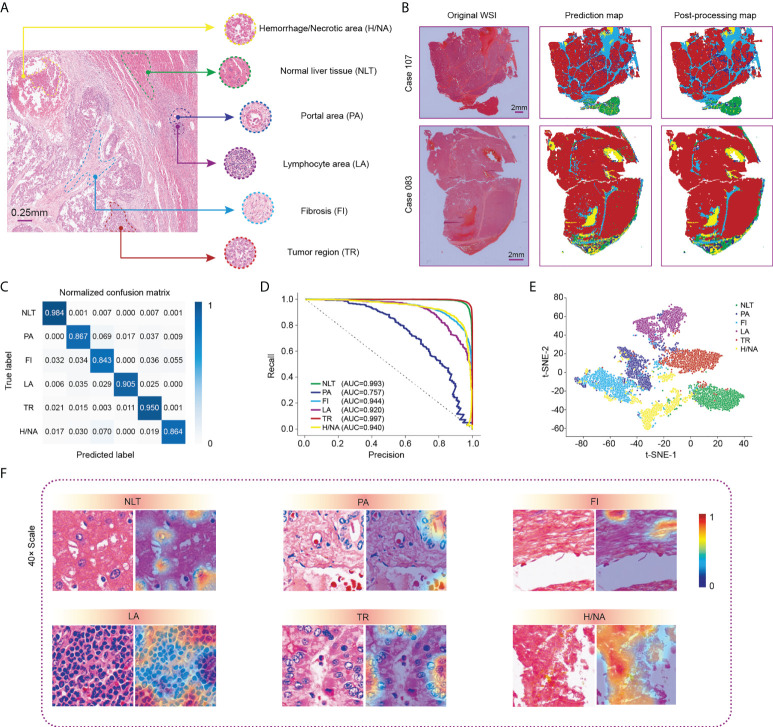
Visualization of WSI classification for HCC tissue in the Zhongshan cohort. **(A)** Six classic categories of HCC tissue, including normal liver tissue (NLT), portal area (PA), fibrosis (FI), lymphocyte area (LA), tumor region (TR) and hemorrhage/necrotic area (H/NA). **(B)** Two representative outputs of the classification network. Red represents TR, green represents NLT, light blue represents FI, dark blue represents PA, purple represents LCA, and yellow represents H/NA. **(C)** Precision-recall curve of category-based sampling. **(D)** Normalized confusion matrix for the classification network. **(E)** t-SNE analysis for visualization of six tissue categories. **(F)** CAM results for visualization of HCC tissues. HCC, hepatocellular carcinoma; CAM, class activation mapping; t-SNE, t-distributed stochastic neighbor embedding.

We first built the category-based model by training a classification network according to HCC tissue. The distribution of HCC tissues in a slide is displayed in **Figure 2A**. Corresponding histological images of the six categories before and after staining standardization are shown in **Supplementary Figures S1A, B**. Representative examples of the raw outputs of the classification network and their postprocessing results by morphology are revealed in **Figure 2B**, with different colors representing distinct tissue components. A visualization of a typical classification map in the TCGA cohort is shown in **Supplementary Figure S1C**.

After comprehensive training, we used the testing dataset to verify the performance of the computation network, which revealed an overall accuracy of 94.17%. Specifically, the accuracy values for normal liver tissue and tumor regions, shown in the confusion matrix (**Figure 2C**), were 0.984 and 0.950, respectively. A precision-recall (PR) curve rather than an ROC curve was applied to evaluate the model to minimize the imbalance. The area under the curve (AUC) values of each tissue type, except for the portal area, in our classification model exceeded 0.920 with the highest in tumor region classification (AUC= 0.997) (**Figure 2D**). **Figure 2E** shows the t-SNE visualization of the classification results. One thousand tiles were randomly selected for each tissue category.

We then used class activation mapping (CAM) (30) of the last convolution layer to visualize the outputs of each tissue category, where redder and bluer heatmaps indicated regions with higher or lower interest, respectively (**Figure 2F**).

### Signature extraction and construction of prognostic scores

Next, we analyzed all 416 WSIs in the training cohort using the classification network and extracted pathological signatures from tiles of each type of HCC tissue. A total of 133 signatures and their coefficients were derived from LASSO Cox analysis (**Supplementary Figure S2**). Univariable Cox analysis of signatures that had a significant impact on RFS is shown in **Supplementary Table S2**.

**Figure 3:**
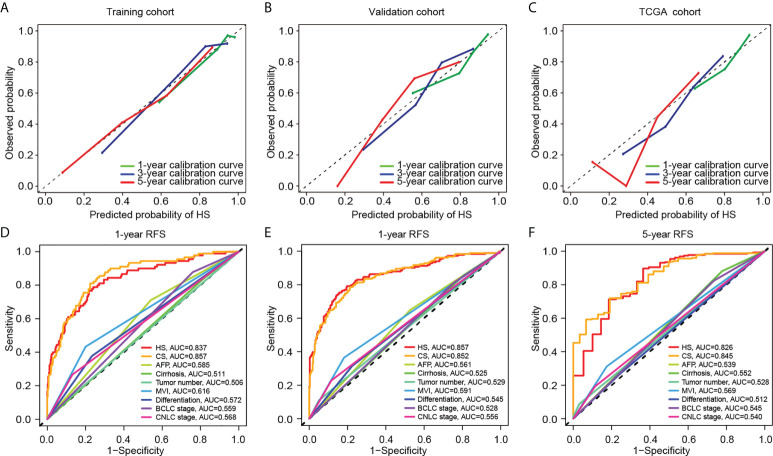
Comparison of predictive performance between two novel models. Upper: The calibration curves for TTR of HS in the training **(A)**, validation **(B)**, and TCGA cohort **(C)**. Down: The ROC curves for 1-year **(D)**, 3-year **(E)** and 5-year **(F)** RFS based on different clinicopathological features and stages. TCGA, The Cancer Genome Atlas; RFS, recurrence-free survival; HS, histological score; CS, combined score; AFP, alpha-fetoprotein; MVI, microvascular invasion; BCLC, Barcelona Clinic Liver Cancer; CNLC, China Liver Cancer; AUC, area under the curve.

HS and CS were then obtained by the sum of products by indexes and coefficients (details are provided in the **Supplementary Methods**). A calibration curve was constructed to evaluate the prediction accuracy. HS showed great concordance between the predicted and observed recurrence probabilities in the training, validation and TCGA cohorts (**Figures 3A** **–** **C**). Similarly, CS performed well in the training and validation cohorts (**Supplementary Figure S3**). To further compare the prediction reliability between the new scores and traditional biomarkers and stages, we carried out ROC curve analysis based on the cases in the training and validation cohorts. In terms of 1-year RFS, the AUC values of HS and CS were 0.837 and 0.857, respectively, much higher than those of clinical indicators such as AFP or liver cirrhosis, and the CNLC or BCLC staging systems (**Figure 3D**). The AUC values of HS and CS reached 0.857 and 0.852 for 3-year RFS prediction and 0.826 and 0.845 for 5-year RFS prediction, respectively (**Figures 3E** **, F**).

### Comparison of HS and CS in predictive accuracy

The C-indexes of HS, CS and clinical signatures were calculated to estimate the possibility of using histological textures as a substitute for clinical indicators. Compared to the clinical signatures, HS and CS presented higher C-indexes in the Zhongshan datasets (**Table 1**). Specifically, the C-indexes of HS were 0.804, 0.739 and 0.708 in the training, validation and TCGA cohorts, respectively. Both HS and CS performed better than clinical signatures in RFS prediction (**Supplementary Figure S4**). Ultimately, ROC curves in the two Zhongshan datasets showed no significant differences between HS and CS for 1-year, 3-year, or 5-year RFS prediction (**Supplementary Figure S5**, **Supplementary Table S3**). The NRI elucidated the quantitative difference between HS and CS in TTR prediction (**Supplementary Figure S6**). Subsequently, the CS model was superior to HS in all three periods with a subtle advantage, but statistical significance was not reached (*p*> 0.1).

**Table 1:** C-indexes of the novel scores.

Cohort	HS	CS	Clinical signatures
Training	0.804 (0.771-0.837)	0.809 (0.777-0.840)	0.653 (0.611-0.695)
Validation	0.739 (0.686-0.792)	0.754 (0.705-0.802)	0.686 (0.630-0.742)
TCGA	0.708 (0.635-0.780)		

∗ Values are presented as C-index (95% confidential interval). HS, histological score; CS, combined score; TCGA, The Cancer Genome Atlas.

### Survival prediction of novel pathological predictors

**Figure 4:**
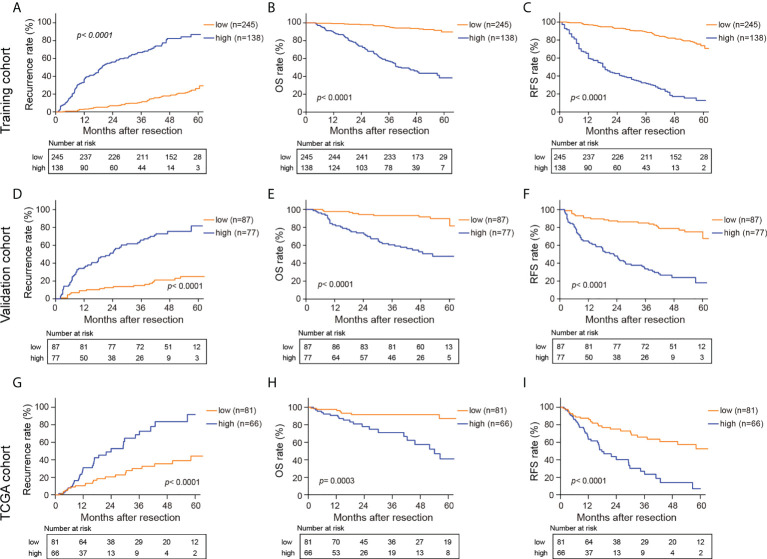
Kaplan-Meier curves for recurrence rate, OS, and RFS in the training **(A–C)**, validation **(D–F)** and TCGA cohorts **(G–I)** based on HS. HS, histological score; OS, overall survival; RFS, recurrence free survival.

Optimal cutoff values for HS and CS were determined using the “survminer” package (31). All the patients were then divided into a high-risk group (HS> -0.1605954, CS> 3.417892) and a low-risk group (HS≤ -0.1605954, CS≤ 3.417892). **Figure 4** and **Supplementary Figure S7** depict the survival curves of HS and CS, respectively. Generally, patients in the high-risk group from the three cohorts faced notable susceptibility to postoperative recurrence and poorer survival.

### Prognostic predictors of RFS in early-stage HCC

We performed Cox proportional hazards regression analysis to explore the independent predictors for RFS in the training and validation cohorts (**Table 2**). Eleven candidates were proven to be significant in the univariable analysis and were then evaluated with multivariable Cox regression. The multivariable analysis revealed that MVI (HR= 1.459, 95% CI: 1.094-1.948) and HS (HR= 4.054, 95% CI: 3.397-4.838) were significant indicators.

**Table 2:** Cox proportional hazards regression model showing the association of variables with RFS.

Variables	Univariate analysis			Multivariate analysis		
	HR	95%CI	*p* value	HR	95%CI	*p* value
HBsAg (yes/no)	1.487	(1.030-2.146)	0.034	1.066	(0.728-1.560)	0.743
Albumin, g/L	0.945	(0.938-0.993)	0.013	0.978	(0.948-1.009)	0.159
AFP (>20/≤20, ng/mL)	1.459	(1.125-1.890)	0.004	1.095	(0.835-1.435)	0.512
GGT, U/L	1.001	(1.000-1.003)	0.045	1.000	(0.999-1.002)	0.516
PT, s	1.131	(1.008-1.269)	0.037	1.047	(0.935-1.174)	0.425
Liver cirrhosis (yes/no)	1.666	(1.136-2.442)	0.008	1.389	(0.922-2.092)	0.116
Tumor number (multiple/single)	1.430	(1.009-2.027)	0.045	1.361	(0.870-2.129)	0.177
Tumor diameter, cm	1.108	(1.059-1.159)	<0.001	1.029	(0.981-1.079)	0.245
MVI (yes/no)	1.931	(1.475-2.527)	<0.001	1.459	(1.094-1.948)	0.010
Tumor differentiation (Edmondson-Steiner grade III-IV/I-II)	1.341	(1.018-1.765)	0.037	1.039	(0.782-1.380)	0.794
Histological score	4.263	(3.616-5.025)	<0.001	4.054	(3.397-4.838)	<0.001

∗Values are presented as HR and 95%CI. HR, hazard ratio; CI, confidence interval; HBsAg, Hepatitis B virus surface antigen; AFP, α-fetoprotein; GGT, γ-glutamyl transpeptidase; PT, prothrombin time; MVI, micro vascular invasion.

### The correlation between HS and clinicopathological characteristics

**Figure 5:**
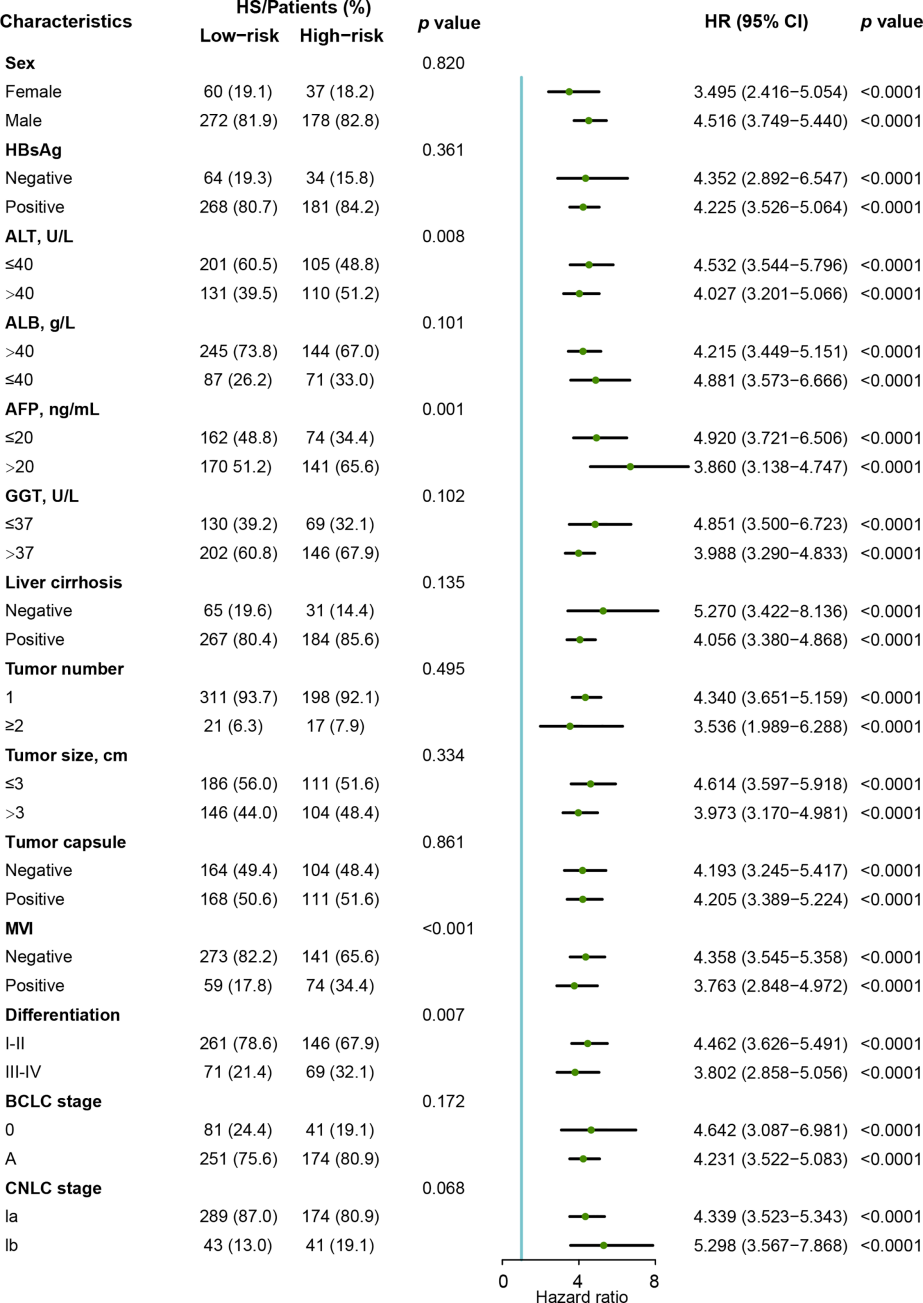
Forest plot of recurrence-free survival based on HS in the Zhongshan cohort. HS, histological score; HR, hazard ratio. ; CI, confidence interval; AFP, α-fetoprotein; ALT, alanine aminotransferase; ALB, albumin; GGT, γ-glutamyl transpeptidase; MVI, micro vascular invasion; CNLC, China Liver Cancer Staging; BCLC, Barcelona Clinic Liver Cancer.

As shown in **Figure 5**, the clinical characteristics and prognostic value of HS were compared in the different risk subgroups, namely, the low-risk group (n= 332) and the high-risk group (n= 215). Compared to patients in the low-risk group, more patients in the high-risk group had elevated AFP levels (65.6% vs 51.2%). Moreover, HCCs in the high-risk group were characterized by poorer tumor differentiation, and a higher proportion of MVI. Seventy-eight (23.5%) patients in the low-risk group experienced recurrence. Conversely, 76.7% of patients in the high-risk group suffered recurrence during the follow-up. In particular, HS was proven to be a prognostic factor in all the subgroups in terms of patients’ clinicopathological characteristics.

The histological markers including proliferation indexes, therapeutic targets, and specific diagnostic markers have been reported to be an efficient indicator for HCC diagnosis and prognosis after resection (32–34). The immunohistochemistry (IHC) and quantitative analysis for six markers was performed in 160 patients (from October 2014 to December 2014). Compared to HS low-risk group, patients in the high-risk group had a higher expression of heat shock protein 70 (HSP70, *p*= 0.01). A slightly higher proportion of Ki-67^+^ cells was observed in the high-risk group although significance was not reached (**Supplementary Figure S8**).

The correlation between pathological subtype and histologic features was also assessed. The results revealed that more patients (12%) in the HS high-risk subgroup were diagnosed with macrotrabecular-massive HCC (MTM-HCC) compared to 5% in the low-risk subgroup (**Supplementary Figure S9**).

### The correlation between HS and the immune microenvironment

**Figure 6:**
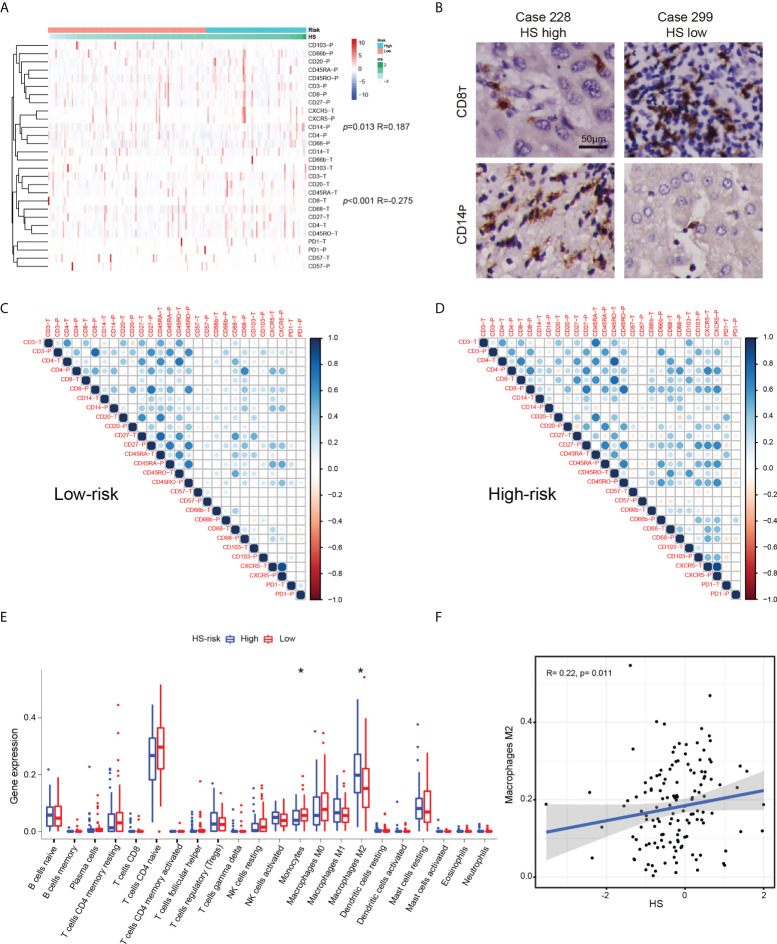
The relationship between HS and immune infiltration condition. **(A)** Heatmap and cluster analysis of the Zhongshan TMA. **(B)** Typical immunohistochemical pictures of CD8_T_ AND CD14_P_. **(C)** The corrplots of immune markers in the HS low-risk group. **(D)** The interaction analysis of immune markers in the HS high-risk group. **(E)** Boxplot of immune cells in the TCGA cohort. Data are compared using Wilcoxon test. *p < 0.05;. **(F)** Correlation analysis between HS and M2macrophages in the TCGA cohort. HS, histological score; TCGA, The Cancer Genome Atlas.

Using TMA data, we examined the expression patterns and distributions of 14 immune markers in 175 HCC patients with early-stage HCC (35). To investigate the relationship between HS and local immune status, we carried out clustering and correlation analysis (**Figure 6A**). We found that HS was positively correlated with the expression of peritumoral CD14 (*p*= 0.013, R= 0.187), but negatively correlated with the infiltration of intratumoral CD8 (p< 0.001, R= 0.275). Typical immunohistochemical images of the two markers are shown in **Figure 6B**. To investigate the interrelation between immune markers, we performed correlation analysis in different risk subgroups of HS (**Figures 6C**, **D**). Compared to the low-risk group, the predominant immune cells in the high-risk group were characterized by CD66-, CD68-, CD103- and CXCR5-positive cells infiltrating both tumoral and peritumoral tissues, which implied that macrophages or neutrophils may play a potential role in the progression of recurrence.

The immune microenvironment of cases in the TCGA datasets was investigated by CIBERSORT algorithm (28). The boxplot revealed significantly higher expression of monocytes and lower expression of M2 macrophages in the HS low-risk subgroup (**Figure 6E**). HS was positively correlated with the infiltration of M2 macrophages (*p*= 0.011, R=0.275, **Figure 6F**). Similar findings suggested an increased interaction among macrophages, dendritic cells and other immune cells based on the corrplots in the TCGA database (**Supplementary Figure S10**).

## Discussion

The emergence of AI has reformed multiple aspects of cancer management. The combination of deep learning and digital WSIs has alleviated the labor in detection and revealed the decent accuracy and efficacy for prognostic models in different solid tumors. The recurrence of early-stage HCC after resection remains a major obstacle in curative treatment. Although multiomics analysis highlighted potential recurrence-related determinants and therapeutic targets (8), its high cost and complexity have hindered its prevalent utilization. Herein, we integrated a neural network with the massive WSIs of HCC patients in BCLC stage 0-A and successfully developed an efficient recurrence prediction index that was prognostic of OS, TTR, and RFS. The novel index was validated in three independent cohorts, demonstrating the generalizability of our approach. By analyzing substantial TMA data, we found that immune infiltration status could potentially provide prognostically valuable information on histological texture.

Recent studies on AI reported novel prognostic models for HCC patients based on pathological images. Saillard et al. established two independent scores using an unsupervised neural network algorithm and attention mechanism according to tumoral or nontumoral annotated tiles (20). Both models showed high accuracy in survival prediction and strong correlations between clinical characteristics. Gao et al. innovatively divided HCC slides into four categories,tumor tissue, normal liver tissue, stroma, and necrosis, and then discriminated the features with high AUC values (21). The study developed and validated an efficient survival prediction model based on a large number of WSIs.

In these two previous studies, pathological signatures were directly generated and extracted by traditional CNNs. CNNs are usually regarded as a “black box”, in which the data are processed through complex computing layers; thus, it is difficult to concretize and interpret the relevant features of samples. In the present research, we manually input abundant signatures of the most relevant tiles, automatically screened the signatures with the recurrence time as the labels *via* LASSO Cox regression, and visually presented the weight of each signature. This procedure bypassed the need for the manual recognition of numerous postprocess tiles. In addition, the image signatures that came from empirical utilization ensured the feasibility (36, 37).

Compared to reported studies, our study has advantages in terms of multicategory training, heatmap visualization, a more accurate prediction of recurrence and multifaceted analysis. HCC is highly heterogeneous with regard to not only genetics or epigenetics but also histology. Despite the classic histological categories mentioned above, the portal area and lymphocyte area are also important structures. Recently, tertiary lymphoid structures were proven to be a favorable factor for prognosis and recurrence status (38), and immune infiltration conditions are becoming more determinant in precision therapy (39). The portal area is a connective tissue among hepatic lobules consisting of branches of the hepatic artery, portal vein and hepatic ducts, in which lymphatic vessels and nerve fibers exist. It was reported that the number of portal areas and inflammation or iron deposition around the portal area were associated with the pathogenesis of HCC (40, 41). Herein, these two structures were originally annotated and trained in the study. The AUC value for the portal area was 0.757, lower than that of the other five tissues, which could be caused by poor structural purity and inadequate amounts. Technically, modified image standardization balanced the color differences among tiles, which raised the overall recognition accuracy for HCC structures up to 94.17%. We also applied the CAM method to visualize the importance of the local structure. As shown in **Figure 2**, cells attracted more attention for recurrence than cell-free areas such as fibrosis and necrosis areas, which made it easier for us to intuitively understand the microscopic information.

Subsequently, we established two recurrence prediction scores *via* LASSO Cox analysis and derived each score through the overall WSIs to maximally preserve the pathological signatures of all sections. Both scores showed great congruence with the recurrence probability and survival. No significant difference was found between HS and CS under NRI analysis, which consequently suggested the feasibility of the potential replacement of important clinical characteristics with pure histological features. In Gao et al’s research, the newly constructed score aimed at OS and its median C-indexes reached 0.731 and 0.713 in two cohorts. Therefore, we specifically targeted recurrence conditions after resection and complemented the recurrence-related information in the TCGA database, which was not included in Gao’s study. In our study, the C-indexes of HS for TTR prediction reached 0.804, 0.739 and 0.708 in the training, validation and TCGA cohorts, respectively.

We compared clinicopathological characteristics between the two risk subgroups. Notably, a larger proportion of MTM-HCC was found in the HS high-risk group, indicating that invisible information processed by deep learning could be explained by the specific texture of tumor cells. In contrast to Saillard et al’s dissected tiles of MTM structures, we analyzed the pathological subtype by a whole slide, which was closer to the definition of MTM-HCC (42). As a rare and highly malignant tumor subtype of HCC, MTM-HCC was proven to be correlated with an increased recurrence risk and poor survival (42, 43). Our study supported this finding. Staining markers for pathological evaluation have gained increasing attention in postoperative management. HSP70 is identified as an upregulated marker in HCC components and performs its role at several points of apoptotic signaling (33). A significant difference was observed in the expression of HSP70 between two risk subgroups, which was consistent with the previous study (44).

The immune microenvironment plays a crucial role in tumor progression and recurrence. Using TMA data, we fully explored the relationship between HS and immune infiltration. CD14-P and CD8-T were shown to be of significance. Our results were supported by a previous study showing that high densities of both CD3(+) and CD8(+) T cells in both the interior and margin were significantly associated with a low rate of recurrence (*p*= 0.007) and prolonged RFS (*p*= 0.002) (45). A recent study reported that a high density of marginal CCR1^+^ CD14^+^ monocytes positively correlated with CCL15 expression and was an independent index for dismal survival (46). Moreover, peritumoral monocytes were found to promote HCC progression by inducing cancer cell autophagy (47), which probably led to the dense expression of CD14-P in patients with high recurrence risk.

From TCGA immune data, we observed that more M2 macrophages were aggregated in the tumor tissues of patients with high HS. This finding was consistent with the prognostic value and tumor biochemical modulation of M2 macrophages (48).

The correlation analysis in both the Zhongshan and TCGA cohorts implied different interaction patterns of immune markers. The stronger interactive effect of macrophages and dendritic cells in the HS high-risk subgroup highlighted the important status of antigen presentation during tumor progression. The result may offer new prospects for further fundamental research.

There are several limitations in our study. First, our training data came from a single institution. There may be image inconsistency in model validation; thus, standardization is an essential step for processing. Second, the patients in both cohorts were predominantly infected with HBV, which reduced the representativity of an extensive HCC population. A more rigorous external validation dataset needs to be validated before routine clinical use. Third, our research carried out deep learning at the histologic level of WSIs. Further study could focus on single-cell discrimination, such as lymphocytes and cancer-associated fibroblasts. Fourth, AI-based study of multiomics sequencing information, more staining markers or images of multicomplex immunofluorescence could be further combined into the prediction model.

## Conclusion

In conclusion, the study proposed an efficient recurrence prediction score for patients with early-stage HCC based on deep learning. The prognostic pathological features identified in digital WSIs composed a computable index to discriminate patients in terms of their relapse risk. The new model derived by weakly supervised training facilitated the classification process of typical HCC tissues, depicted the immune infiltration condition in intratumoral and peritumoral structures, and highlighted the clinical characteristics that were significant to prognosis. Further AI research may pay attention to interpretation at the cellular level and the integration of therapeutic decisions or multiomics sequencing.

## Data availability statement

The raw data supporting the conclusions of this article will be made available by the authors, without undue reservation.

## Ethics statement

The study obtained ethical approval from the Institutional Review Board of Zhongshan Hospital (B2021-611) and complied with the standards of the Declaration of Helsinki. Informed consent was received from each patient before the research.

## Author contributions

(I) Conception and design: W-FQ, M-XT, Y-HS; (II) Administrative support: Y-HS, Y-YH, HZ; (III) Provision of study materials or patients: Y-HS, Y-YH, HZ, JZ, JF; (IV) Collection and assembly of data: W-FQ, M-XT, J-TQ, Y-CG, W-RL, ZT, C-YT, W-AH, X-YL, Z-XW, KQ; (V) Data analysis and interpretation: W-FQ, M-XT, J-TQ; (VI) Manuscript writing: W-FQ, M-XT, J-TQ; (VII) Final approval of manuscript: All authors.

## Funding

This work was supported by grants from the National Natural Science Foundation of China (No.81773067, 81902963, 882073217, 82073218, and 82003084), Shanghai Sailing Program (19YF1407800), Intelligent Chronic Disease Management System Based on Edge and Cloud Computing Cooperation (2020-002), Shanghai Municipal Science and Technology Major Project (Grant No. 2018SHZDZX05). Shanghai Municipal Key Clinical Specialty. CAMS Innovation Fund for Medical Sciences (CIFMS) (2019-I2M-5-058). National Key R&D Program of China (2020YFE0202200, 2018YFF0301102 and 2018YFF0301105).

## Conflict of interest

The authors declare that the research was conducted in the absence of any commercial or financial relationships that could be construed as a potential conflict of interest.

## Publisher’s note

All claims expressed in this article are solely those of the authors and do not necessarily represent those of their affiliated organizations, or those of the publisher, the editors and the reviewers. Any product that may be evaluated in this article, or claim that may be made by its manufacturer, is not guaranteed or endorsed by the publisher.
