# Supplementary material for: Exploring pathological signatures for predicting the recurrence of early-stage hepatocellular carcinoma based on deep learning
Source: Front Oncol. 2022 Aug 19;12:968202. doi: 10.3389/fonc.2022.968202 (PMC9439660; doi:10.3389/fonc.2022.968202)
Supplement: Supplementary file 1 [file DataSheet_1.docx]

**Supplementary Methods**

***Extension of OTSU methods***

*Treatment of special cases:* In the process of tissue extracting, some patients suffered fatty liver disease, in which blank bubbles existed in the foreground area of the WSIs. Morphological “WhiteTopHat”[1] was applied to fill the bubbles to solidify the foreground area after OTSU[2].

*Details of extract tiles:* First, we used the OTSU method to extract the foreground area to generate segmentation masks in the minimum dimension level using the OpenSlide library (<https://openslide.org/>). Next, for each effective pixel in the mask, we obtained the position of the pixel in the original image (40x magnification) according to the coordinate transformation. Meanwhile, we extracted tiles in the 299ⅹ299-pixel windows at a magnification of 40x under sliding steps of 299. Only the tiles with corresponding foreground part greater than 30% would be retained.

***Data balancing***

Tiles of six HCC categories were processed in two ways: When the number of tiles was insufficient, we performed data augmentation by random horizontal and vertical flipping, with rotation range 0-30 degrees, shear range 0-0.2 possible values, width and height shift range 0-0.2 possible values. The filling mode was selected as “reflecting”. At the same time, the tiles in which white background occupied more than 75% were deleted to reduce the interference of useless data. Superfluous tiles were randomly extracted to ensure the consistency in the number of tiles for each category.

***Standardization of TCGA diagnostic slides***

We used the traditional Reinhard algorithm[3] to perform staining standardization initially. However, distortion existed in the tiles in which less chromatism or no nucleus was stained in cells. Through experiments, we found that such problem could be alleviated by choosing larger tiles. We cut 5000×5000 central images from each WSI, instead of all the tiles, to calculate mean and standard deviation, so as to reduce the adverse impact brought by calculating tiles during the staining standardization.

We chose a tile from Zhongshan WSIs as the target image, and cut central image as the representative image of each WSI. We transformed the original, target and representative image’s color space from RGB to CIELab (Lab). Then, the original image preformed staining standardization in Lab color space. The calculation formula for original image was as follows:

$$l_{original}=\frac{l_{original}-\bar{l}_{wsi\_represent}}{\hat{l}_{wsi\_represent}}\hat{l}_{target}+\bar{l}_{target}$$

$$\alpha_{original}=\frac{\alpha_{original}-\bar{\alpha}_{wsi\_represent}}{\hat{\alpha}_{wsi\_represent}}\hat{\alpha}_{target}+\bar{\alpha}_{target}$$

$$\beta_{original}=\frac{\beta_{original}-\bar{\beta}_{wsi\_represent}}{\hat{\beta}_{wsi\_represent}}\hat{\beta}_{target}+\bar{\beta}_{target}$$

$\bar{l}$, $\bar{\alpha,}\bar{\beta}$ is the mean value of three channels in Lab color space, $\hat{l}$，$\hat{\alpha}$, $\hat{\beta}$ is the standard deviation of three channels in Lab color space. The stained original image was transformed back to RGB color space.

***Classification network***

*Implementation details:* We used the 116 annotated WSIs for establishing the multi-classification model. After preprocessing, all the tiles, intercepted from WSIs, were input into the network. The parameters of the Inception-V3[4] were random initialized without pretrained. We used the categorical cross-entropy between the predicted probability and the real label as loss, and the parameters to stochastic gradient descent included momentum of 0.9 and weight decay of 1E-2 with batch-size 32. The initial learning rate was 1E-3, and multiplied by 0.1 when the evaluation index was not improving in three consecutive epochs, minimum reduced to 1E-6. The training process was iterated 50 epochs.

*Generating classification maps:* We used the trained classification network to recognize all the WSIs for each patient. The classification results of all the tiles which corresponded to pixels were integrated into the preliminary classification maps.

We modified the spatial correlation between adjacent tiles through morphology to improve the consistency. The hemorrhage/necrotic area and lymphocytes concentration area were clustered and petty in WSIs, hence we removed small holes with area less than 64 and objects with area less than 4, setting connectivity parameter as 1. There were usually small cavities in the tumor area, portal area and fibrosis. We used the closing method rather than removing the holes to eliminate disturbance. The kernels with radius 1 was used as the parameters in the closing method. Finally, all the modified tissue maps were integrated to obtain the classification map. The morphological methods were performed using “scikit-image SciKit” (toolkit for SciPy).

***Pathological signature extraction in WSIs and clinical characteristics***

*Implementation details:* After computational prediction, ten tiles with the highest prediction probability of each type in the WSI were selected for signature extraction. Each tile obtained 107 original pathological signatures (<https://pyradiomics.readthedocs.io/en/latest/features.html>). Next, in the individual HCC tissue type, the mean, standard deviation, median and decile of the corresponding tiles’ signatures were calculated as the final candidates. Moreover, the proportion of the area in each tissue type was counted as the supplementary signatures. As a result, each WSI had a total of 2574 pathological signatures.

*Clinical characteristics:* After a detailed history and a complete physical examination, blood was taken from the patients for hepatitis B surface antigen (HBsAg), serum albumin (ALB), aspartate transaminase (ALT), α-glutamyl transferase (GGT), alkaline phosphatase (ALP), α-fetoprotein (AFP), carbohydrate 19-9 (CA 19-9), and carcinoembryonic antigen (CEA). Clinicopathological features were derived from the pathological reports including liver cirrhosis, tumor number, tumor size, capsule, micro vascular invasion (MVI), lymphoid metastasis and differentiation.

***Feature selection of Lasso Cox analysis***

We used LASSO Cox regression model[5] to preliminarily select prognostic features by using “glmnet” R package. The pathological signatures and clinical characteristics were input as x matrix while the recurrence status and recurrence time were response y. Harrel’s concordance measure was used as loss for 10-folds cross-validation. We chose the optimal lambda that resulted to the minimum cross-validated error. The lambda was input into the fitted model to derive indexes and coefficients of no-zero features. HS and CS were obtained by the sum of products by corresponding indexes and coefficients.

The formulas of HS and CS were as follows:

x1 = "(-0.122016800369092*m0_shape_Flatness_mean)+(7.75700144152009e-15*m0_shape_MeshVolume_mean)+(-0.104021271905972*m0_glcm_ClusterShade_val)+(-0.00728318533363779*m0_glcm_DifferenceVariance_val)+(0.0634685972678304*m0_glcm_MCC_val)+(-0.0720479395240757*m0_gldm_GrayLevelNonUniformity_val)+(-0.007441131169821*m0_firstorder_RobustMeanAbsoluteDeviation_median)+(0.0237957282652736*m0_glrlm_HighGrayLevelRunEmphasis_median)+(-0.0242529667308698*m0_glszm_LowGrayLevelZoneEmphasis_median)+(-0.0137204628864997*m0_ngtdm_Contrast_median)+(0.0843469888719412*m0_gldm_DependenceNonUniformity_median)+(-0.026765044562175*m0_firstorder_Median_deciles)+(-0.0354914627269978*m0_firstorder_RootMeanSquared_deciles)+(-0.0573236766580267*m0_glcm_DifferenceEntropy_deciles)+(2.69962382794311e-05*m0_glcm_Imc2_deciles)+(-0.0345841446130225*m0_glcm_JointAverage_deciles)+(0.102764310269742*m0_glszm_LargeAreaHighGrayLevelEmphasis_deciles)+(0.0428562349015899*m0_glszm_SizeZoneNonUniformity_deciles)+(-0.0301566371871564*m0_gldm_LargeDependenceHighGrayLevelEmphasis_deciles)+(-0.046299665381274*m1_firstorder_90Percentile_val)+(0.000839119908059214*m1_firstorder_Maximum_val)+(-0.0829140955441657*m1_glcm_InverseVariance_val)+(-0.0378819905968139*m1_glrlm_GrayLevelVariance_val)+(-0.147676581527489*m1_glrlm_ShortRunLowGrayLevelEmphasis_val)+(9.86106492097824e-06*m1_glszm_LargeAreaLowGrayLevelEmphasis_val)+(0.033900989497138*m1_glszm_SmallAreaHighGrayLevelEmphasis_val)+(-0.0453632949088534*m1_ngtdm_Complexity_val)+(-0.0202076151839154*m1_gldm_SmallDependenceLowGrayLevelEmphasis_val)+(0.015696516658804*m1_firstorder_Minimum_median)+(-0.0389092509224935*m1_glszm_SmallAreaEmphasis_median)+(0.318539806931387*m1_glszm_SmallAreaLowGrayLevelEmphasis_median)+(-0.109398600534323*m1_ngtdm_Busyness_median)+(0.0591279489202919*m1_firstorder_Energy_deciles)+(0.144189416420948*m1_firstorder_Median_deciles)+(0.00369010277505741*m1_firstorder_TotalEnergy_deciles)+(0.0383076582570865*m1_glcm_Idm_deciles)+(0.0195905164383039*m1_glrlm_RunEntropy_deciles)+(-0.0702692563059623*m1_glszm_SmallAreaEmphasis_deciles)+(-0.0923453799760962*m2_glcm_Imc2_mean)+(0.00342702717007133*m2_glszm_GrayLevelNonUniformity_mean)+(-0.00128111192500525*m2_glszm_LargeAreaEmphasis_mean)+(0.0154366405793262*m2_glszm_SizeZoneNonUniformity_mean)+(0.00135510273373574*m2_firstorder_Maximum_val)+(-0.0381878146468702*m2_glcm_Imc2_val)+(-0.00047769019818483*m2_glszm_GrayLevelVariance_val)+(-0.00174051524257116*m2_glszm_LowGrayLevelZoneEmphasis_val)+(0.0263128989921181*m2_glszm_SizeZoneNonUniformity_val)+(0.0325931785410028*m2_gldm_DependenceVariance_val)+(0.0058891902286527*m2_gldm_LargeDependenceLowGrayLevelEmphasis_val)+(0.000278241507637323*m2_gldm_DependenceVariance_median)+(0.0186964378939344*m2_gldm_LargeDependenceLowGrayLevelEmphasis_median)+(0.0642797873239336*m2_gldm_SmallDependenceLowGrayLevelEmphasis_median)+(0.00463253863909167*m2_firstorder_Uniformity_deciles)+(0.0102635574852423*m2_glcm_Idn_deciles)+(0.00570419161862622*m2_glrlm_RunEntropy_deciles)+(-0.00642658710799511*m2_glszm_LargeAreaLowGrayLevelEmphasis_deciles)+(0.0052230704895731*m2_gldm_GrayLevelNonUniformity_deciles)+(-0.0150816804821197*m2_gldm_SmallDependenceHighGrayLevelEmphasis_deciles)+(0.0275087706706102*m3_firstorder_Minimum_mean)+(-0.0727078544702491*m3_firstorder_Range_mean)+(0.262108075854777*m3_glcm_MaximumProbability_mean)+(0.0963846481219106*m3_glrlm_GrayLevelNonUniformity_mean)+(-0.0240827277704738*m3_glrlm_RunEntropy_mean)+(0.0808465990105737*m3_glszm_SmallAreaLowGrayLevelEmphasis_mean)+(0.101730054217782*m3_gldm_DependenceVariance_mean)+(-0.0641659604047439*m3_glszm_ZoneEntropy_val)+(-0.156128634477967*m3_ngtdm_Strength_val)+(0.0480761327349284*m3_gldm_DependenceVariance_val)+(-0.0158504298440178*m3_glszm_HighGrayLevelZoneEmphasis_median)+(0.239822567610647*m3_glszm_SizeZoneNonUniformity_median)+(-0.0854227134853446*m3_ngtdm_Strength_median)+(-0.374990042858843*m3_firstorder_Variance_deciles)+(-0.0288118947062985*m3_shape_Maximum2DDiameterSlice_deciles)+(0.0412986530691085*m3_glcm_InverseVariance_deciles)+(0.0545821784331602*m3_ngtdm_Busyness_deciles)+(-0.0166470771076297*m4_firstorder_RobustMeanAbsoluteDeviation_mean)+(-5.95091408028028e-05*m4_shape_Elongation_val)+(-0.000327263427893282*m4_shape_Maximum3DDiameter_val)+(-0.0340888177118691*m4_glrlm_GrayLevelNonUniformity_val)+(-0.145572056812165*m4_glrlm_RunEntropy_val)+(-0.00520738835700461*m4_glszm_SmallAreaLowGrayLevelEmphasis_val)+(0.0559264206173697*m4_glcm_JointEntropy_median)+(0.0814362756256773*m4_glrlm_RunPercentage_median)+(-0.107068836693169*m4_glszm_SmallAreaLowGrayLevelEmphasis_median)+(-0.0446803057494133*m4_firstorder_Kurtosis_deciles)+(0.0735869457136152*m4_firstorder_Variance_deciles)+(0.0905796709524612*m4_glcm_SumEntropy_deciles)+(-0.00682601410330697*m4_glrlm_RunEntropy_deciles)+(0.0422734191404945*m4_glrlm_ShortRunLowGrayLevelEmphasis_deciles)+(0.121043709580541*m4_glszm_SizeZoneNonUniformity_deciles)+(-0.0615050877519192*m4_gldm_DependenceNonUniformity_deciles)+(-1.20400145051144e-07*m4_gldm_DependenceNonUniformityNormalized_deciles)+(-0.0996393035747283*m4_gldm_SmallDependenceHighGrayLevelEmphasis_deciles)+(-6.00690899626568e-07*m5_shape_VoxelVolume_mean)+(-0.123237240690278*m5_glcm_Idmn_mean)+(-0.0885574591978988*m5_shape_Elongation_val)+(-0.0464775090572624*m5_glrlm_LowGrayLevelRunEmphasis_val)+(-0.0902622106981713*m5_glrlm_ShortRunEmphasis_val)+(-0.129049672569143*m5_glrlm_ShortRunLowGrayLevelEmphasis_val)+(0.120089429752362*m5_glszm_GrayLevelNonUniformityNormalized_val)+(0.0882845789655749*m5_ngtdm_Strength_val)+(-0.0684896372349365*m5_gldm_DependenceEntropy_val)+(-0.0128537799943673*m5_gldm_LargeDependenceLowGrayLevelEmphasis_val)+(0.44077193209468*m5_firstorder_Minimum_median)+(-5.36156738191924e-06*m5_shape_Elongation_median)+(0.109782332532557*m5_glrlm_LongRunEmphasis_median)+(0.0377897868976266*m5_glrlm_RunEntropy_median)+(0.138719389519223*m5_glrlm_ShortRunLowGrayLevelEmphasis_median)+(-0.0126701800853986*m5_glszm_SmallAreaHighGrayLevelEmphasis_median)+(-0.146411819724686*m5_ngtdm_Strength_median)+(-0.0072636906885224*m5_firstorder_90Percentile_deciles)+(0.0553896879722332*m5_glcm_Correlation_deciles)+(0.0769298932491218*m5_glrlm_GrayLevelNonUniformity_deciles)+(-0.00566567479165237*m5_glrlm_ShortRunHighGrayLevelEmphasis_deciles)+(0.0283413608763572*m5_glszm_ZonePercentage_deciles)+(0.0864353494096385*m5_ngtdm_Contrast_deciles)+(0.0972885882371829*m5_gldm_DependenceVariance_deciles)+(-0.761630488278793*pred_0)+(0.53192078736907*pred_1)"

x2 = "(-0.00076463928834012*Sex)+(0.00727032956750945*Age)+(0.298169325397803*HBsAg)+(0.000684242884045703*ALT)+(-0.0152305660276121*ALB)+(0.275821849545985*AFP.CATE)+(0.000627552283166938*GGT)+(0.443175586385359*Liver_Cirrhosis_SPSS)+(0.146759389518157*Tumor_Number_Database)+(0.0837535224678497*Tumor_Size_Corrected)+(-0.033596379247241*Capusle_SPSS)+(0.541341012706675*Vascualr_Invasion_Corrected)+(0.635932388065421*lymphoid)+(0.0743711973866074*Differentiation_SPSS)"

HS = x1

Clinical signatures=x2

CS = x1+x2

***Hardware and software***

Classification network was trained using Keras 2.2.4 on servers equipped with one GTX 1080ti GPU cards. Image data from Zhongshan Hospital was scanned using KF-PRO-120. WSIs were read using OpenSlide. The image extraction, preprocessing, deep-learning model building, feature extraction and integration were performed using Python 3.6. Lasso Cox regression and other statistical analysis was performed using R-statistics 3.6.3 (R Foundation, Vienna, Austria).

1. Dougherty E, Lotufo RA: *Hands-On Morphological Image Processing.* Hands-on Morphological Image Processing; 2003.

2. Otsu N: **A threshold selection method from gray-level histograms.** *IEEE transactions on systems, man, and cybernetics* 1979, **9:**62-66.

3. Reinhard E, Adhikhmin M, Gooch B, Shirley P: **Color transfer between images.** *IEEE Computer graphics and applications* 2001, **21:**34-41.

4. Szegedy C, Vanhoucke V, Ioffe S, Shlens J, Wojna Z: **Rethinking the inception architecture for computer vision.** In *Proceedings of the IEEE conference on computer vision and pattern recognition*. 2016: 2818-2826.

5. Tibshirani R: **The LASSO method for variable selection in the Cox Model.** *Statistics in Medicine* 1997, **16:**385-395.
