# Supplementary material for: Exploring pathological signatures for predicting the recurrence of early-stage hepatocellular carcinoma based on deep learning
Source: Front Oncol. 2022 Aug 19;12:968202. doi: 10.3389/fonc.2022.968202 (PMC9439660; doi:10.3389/fonc.2022.968202)
Supplement: Supplementary file 2 [file DataSheet_2.docx]

**Supplementary figures**


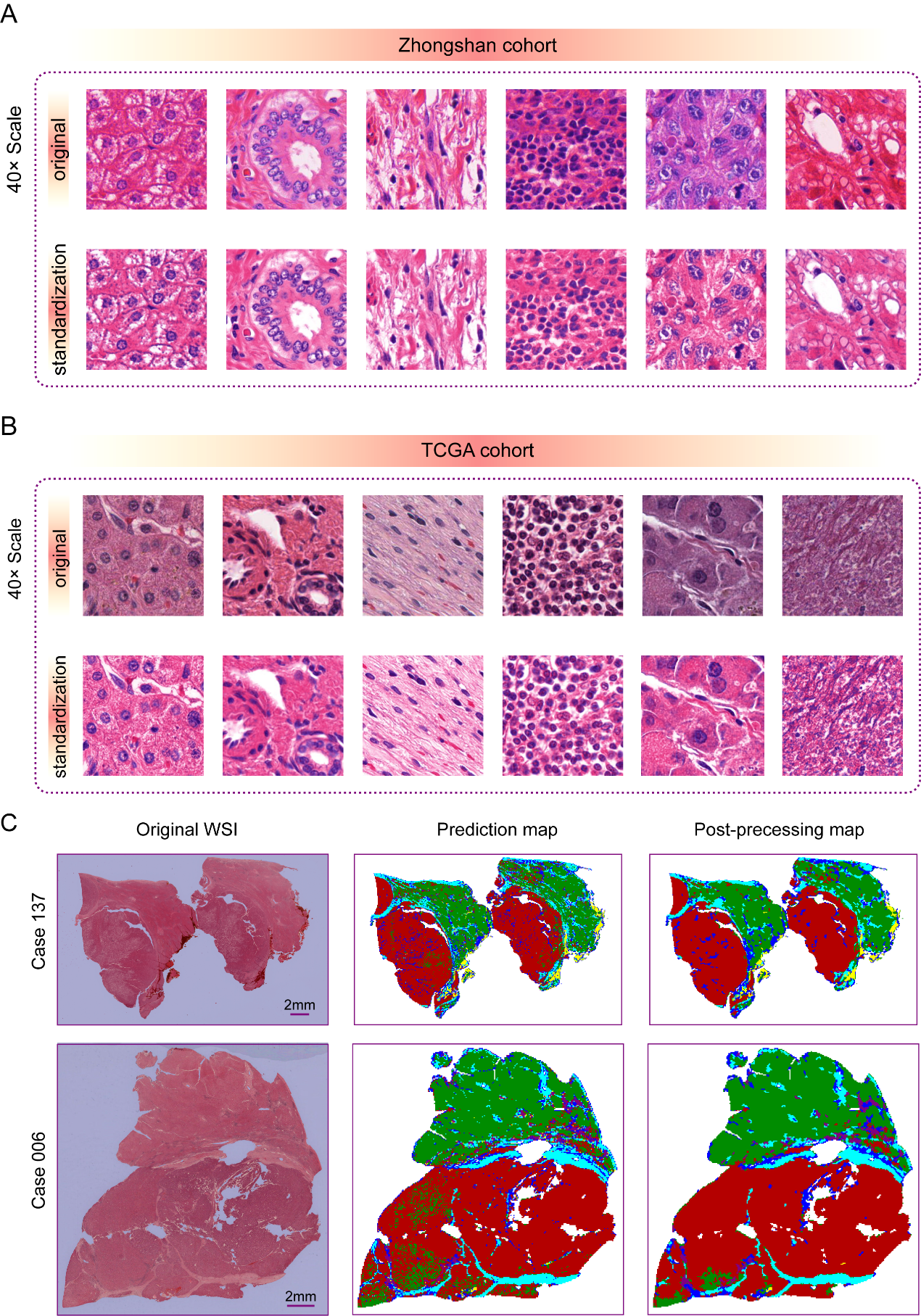


**Figure S1.** The standardization results of six categories of HCC tissue in the (A) Zhongshan cohort and (B) TCGA cohort. (C) The visualization of classification map in the TCGA cohort.


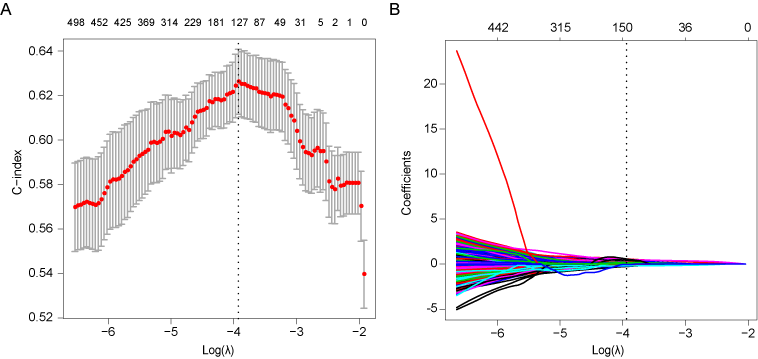


**Figure S2.** The LASSO-Cox analysis of recurrence-related signatures. (A) C-indexes determines the optimal λ value. (B) The λ value determines the nonzero coefficients. LASSO, least absolute shrinkage and selection operator.


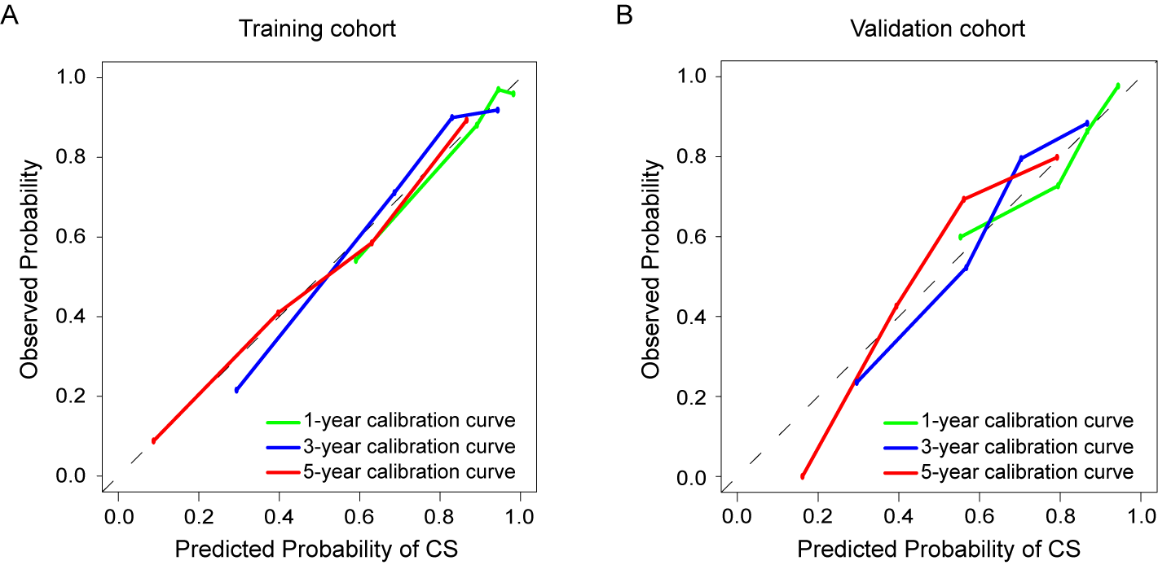


**Figure S3.** The calibration curves of CS in the (A) training cohort and (B) validation cohort. CS, combined score.


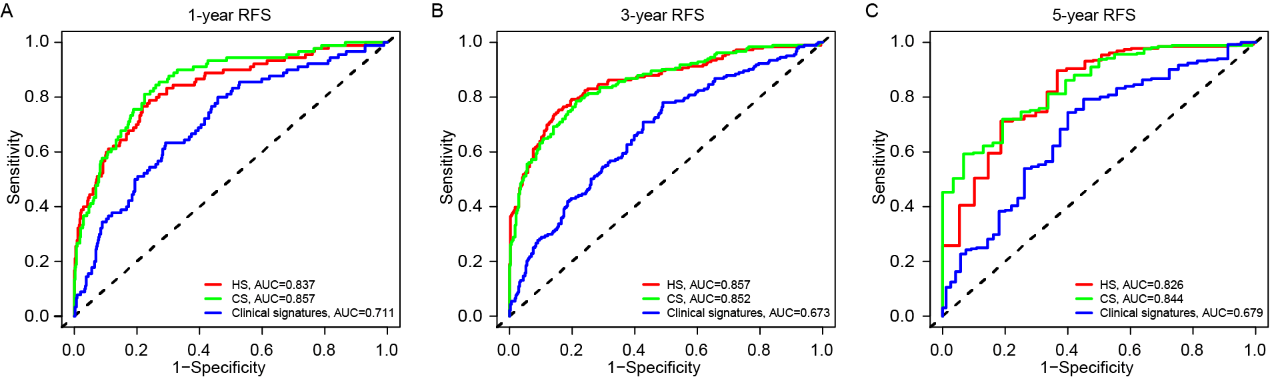


**Figure S4.** The ROC curves of novel scores for predicting 1-year RFS (A), 3-year RFS (B) and 5-year RFS (C). HS, histological score; CS, combined score; ROC, receiver operation characteristic; RFS, recurrence free survival.


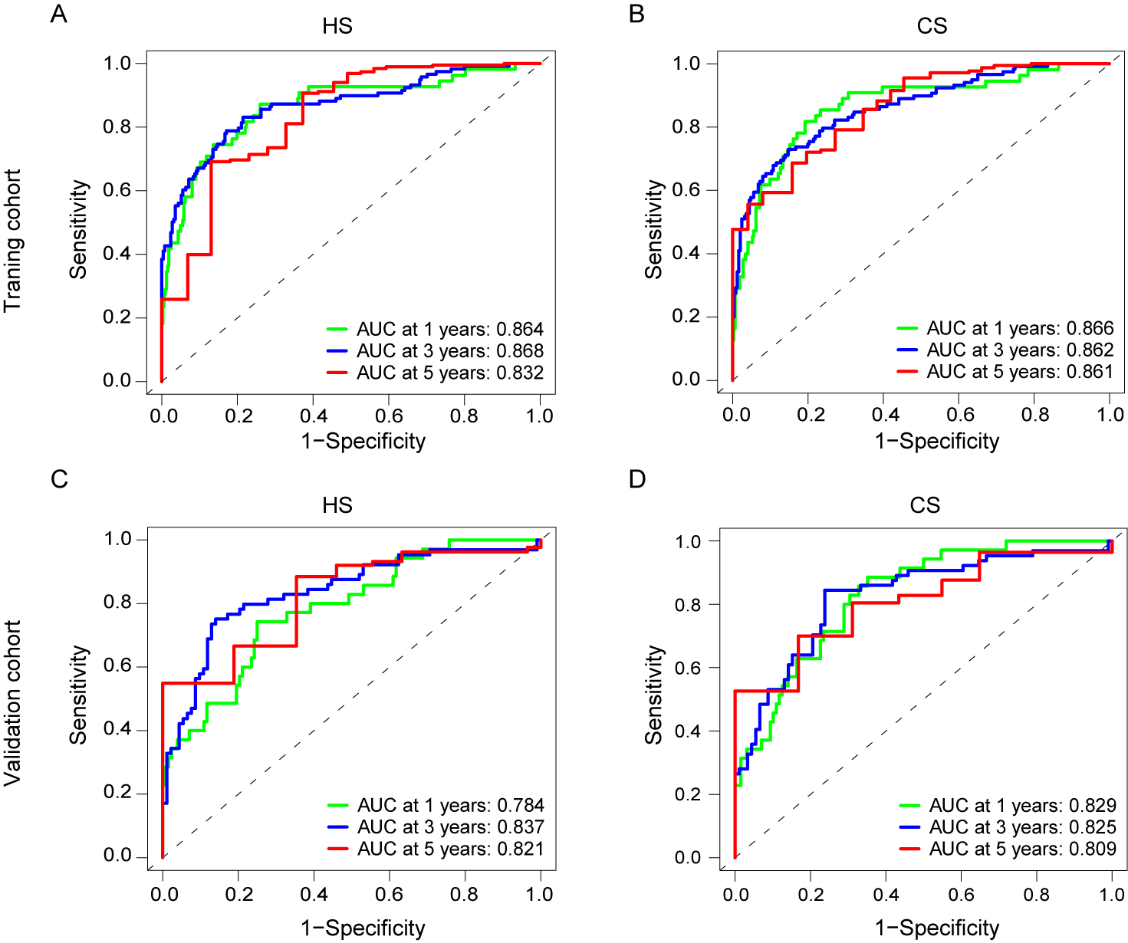


**Figure S5.** The comparison between HS and CS based on the ROC curves in the training cohort (A-B) and validation cohort (C-D). HS, histological score; CS, combined score; ROC, receiver operation characteristic.


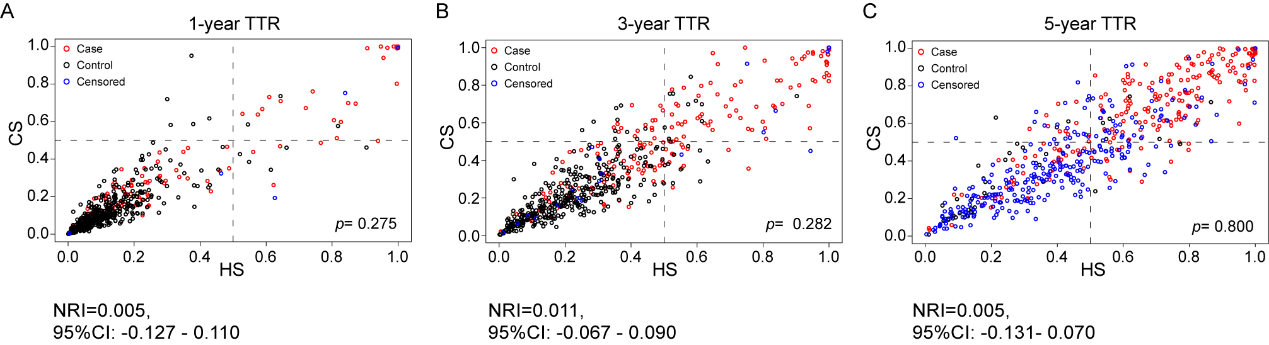


**Figure S6.** The NRI comparison between HS and CS based on the 1-year TTR(A), 3-year TTR (B) and 5-year TTR (C). NRI, net reclassification improvement; HS, histological score; CS, combined score; TTR, time to recurrence.


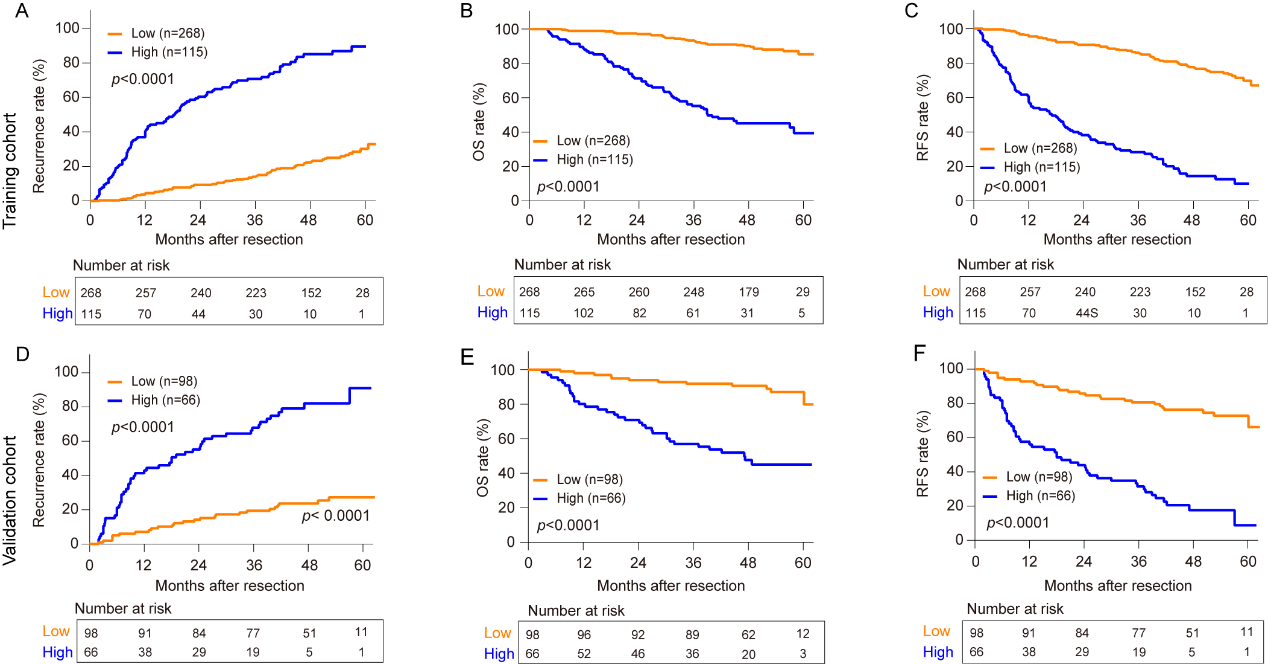


**Figure S7.** Kaplan-Meier curves for recurrence rate, recurrence-free survival, and overall survival in the training and validation cohorts based on CS. CS, combined score.


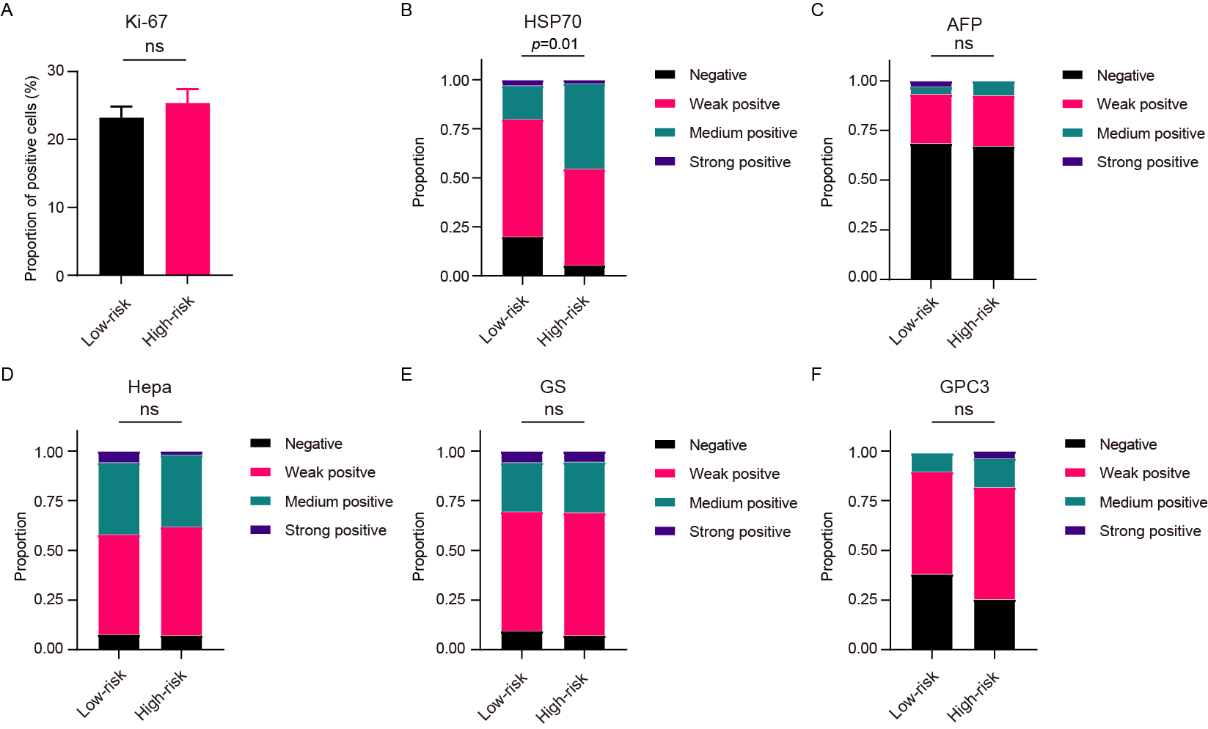


**Figure S8.** The correlation between HS and expression of histological staining markers. (A) The comparison for the proportion of Ki-67^+^ tumor cells between HS low-risk and high-risk subgroups. The comparison for the expression of HSP70 (B), AFP (C), Hepa (D), GS (E), and GPC3 (F) between HS low-risk group and high-risk group. HS, histological score. HSP70, heat shock protein 70; AFP, alpha-fetoprotein; GS, glutamine synthetase; GPC3, glypican-3.


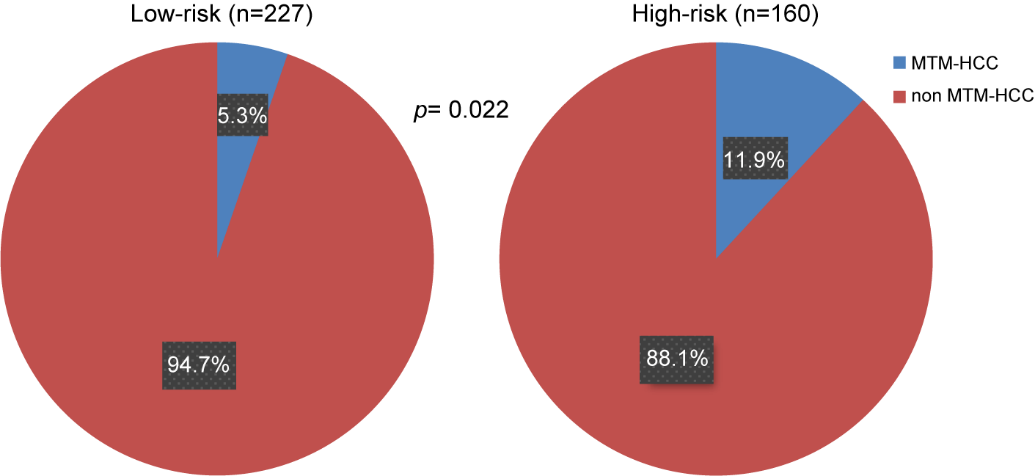


**Figure S9.** The pie charts of MTM-HCC constitution in different risk groups. MTM-HCC, macrotrabecular-massive hepatocellular carcinoma.


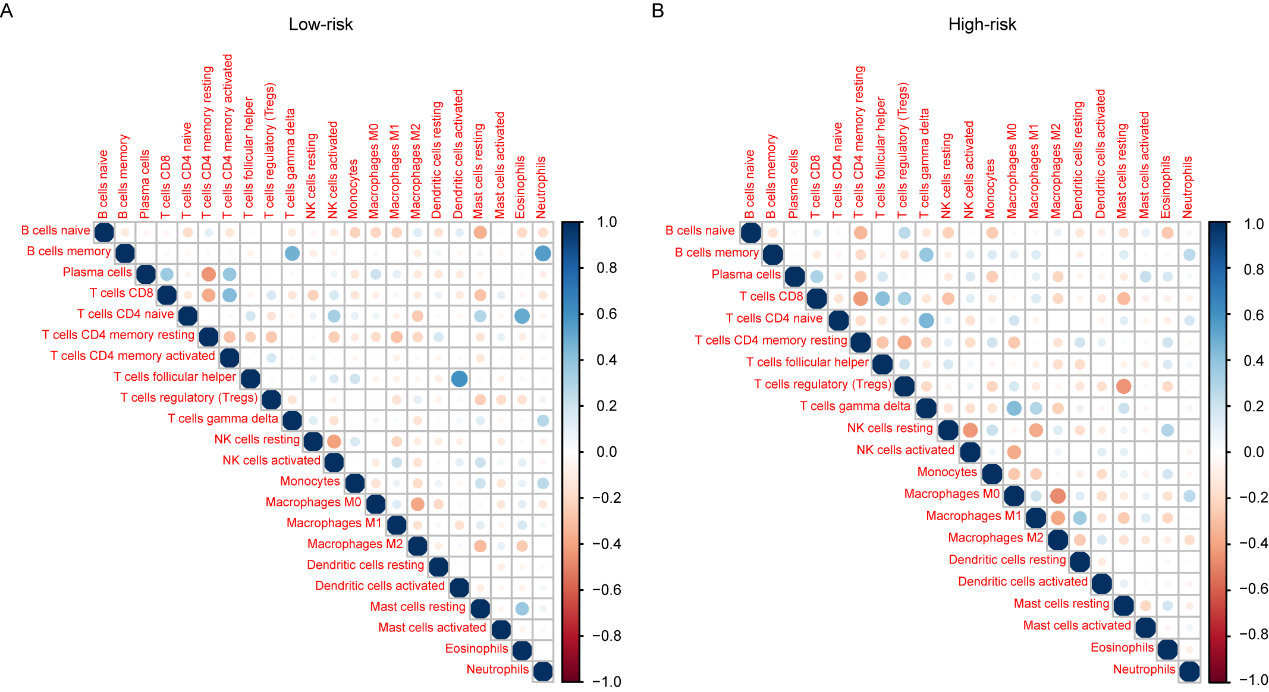


**Figure S10.** The corrplots of immune cells infiltration in the HS low-risk group (A) and HS high-risk group (B) based on TCGA database. TCGA, The Cancer Genome Atlas; HS, histological score.
