# Supplementary material for: Exploring pathological signatures for predicting the recurrence of early-stage hepatocellular carcinoma based on deep learning
Source: Front Oncol. 2022 Aug 19;12:968202. doi: 10.3389/fonc.2022.968202 (PMC9439660; doi:10.3389/fonc.2022.968202)
Supplement: Supplementary file 3 [file Table_1.docx]

**Table S1.** **Demographic, clinical, and tumor characteristics of patients with early-stage HCC.**

| Patient demographics | Training cohort (n=383) | Validation cohort (n=164) | *P* value |
| --- | --- | --- | --- |
| Age, yr | 53(46, 62) | 55(48, 62) | 0.367 |
| Sex (male), n (%) | 310(80.9) | 140(85.4) | 0.225 |
| HBsAg (positive), n (%) | 308(80.4) | 141(86.0) | 0.144 |
| Anti-HCV (positive), n (%) | 8(2.1) | 1(0.6) | 0.291 |
| Liver cirrhosis, yes (%) | 310(80.9) | 141(86.0) | 0.178 |
| AFP (＞20ng/mL), n (%) | 213(55.6) | 98(59.8) | 0.397 |
| Albumin, g/L | 42.4(40.0, 46.0) | 43.0(40.0, 47.0) | 0.333 |
| Bilirubin, μmol/L | 14.0(10.2, 17.7) | 14.1(10.0, 18.1) | 0.237 |
| ALT, U/L | 37.0(24.3, 53.0) | 38.0(26.3, 57.8) | 0.384 |
| GGT, U/L | 46.0(30.0, 76.3) | 47.6(29.0, 75.8) | 0.504 |
| PT, s | 11.6(11.1, 12.3) | 11.7(11.2, 12.4) | 0.699 |
| Tumor number, n (%) |  |  | 0.357 |
| 1 | 353(92.2) | 156(95.1) |  |
| 2 | 26(6.8) | 6(3.7) |  |
| 3 | 4(1.0) | 2(1.2) |  |
| Tumor diameter, cm | 3.0(2.0, 4.0) | 3.0(2.0, 4.5) | 0.493 |
| Tumor capsule (positive), n (%) | 194(50.7) | 85(51.8) | 0.852 |
| Tumor differentiation, n (%) |  |  | 0.088 |
| I-II | 293(76.5) | 114(69.5) |  |
| III-IV | 90(23.5) | 50(30.5) |  |
| Batts-Ludwig hepatitis grade |  |  | 0.280 |
| G0 | 8(2.1) | 5(3.0) |  |
| G1 | 84(21.9) | 30(18.3) |  |
| G2 | 130(33.9) | 61(37.2) |  |
| G3 | 150(39.2) | 58(35.4) |  |
| G4 | 11(2.9) | 10(6.1) |  |
| MVI (yes), n (%) | 89(23.2) | 44(26.8) | 0.385 |
| Lymphoid metastasis (yes), n (%) | 1(0.3) | 0(0.0) | 1.000 |
| CNLC, n (%) |  |  | 0.440 |
| Ia | 321(83.8) | 142(86.6) |  |
| Ib | 62(16.2) | 22(13.4) |  |
| BCLC, n (%) |  |  | 0.738 |
| 0 | 84(21.9) | 38(23.2) |  |
| A | 299(78.1) | 126(76.8) |  |

^∗^Values are presented as no. (%) or median (Q1, Q3). HBsAg, Hepatitis B virus surface antigen; HCV, hepatitis C virus; AFP, α-fetoprotein;; ALT, alanine aminotransferase; GGT, γ-glutamyl transpeptidase; PT, prothrombin time; MVI, micro vascular invasion; CNLC, China Liver Cancer Staging; BCLC, Barcelona Clinic Liver Cancer.
