# Supplementary material for: Exploring pathological signatures for predicting the recurrence of early-stage hepatocellular carcinoma based on deep learning
Source: Front Oncol. 2022 Aug 19;12:968202. doi: 10.3389/fonc.2022.968202 (PMC9439660; doi:10.3389/fonc.2022.968202)
Supplement: Supplementary file 4 [file Table_2.docx]

**Table S2. Univariable Cox regression model showing the association of pathological signatures with RFS in the Zhongshan cohort.**

| Pathological signatures | Univariate analysis | | |
| --- | --- | --- | --- |
|  | HR | 95%CI | *p* value |
| m0_glcm_ClusterShade_val | 0.692 | (0.556-0.863) | 0.001 |
| m0_glcm_JointAverage_deciles | 0.848 | (0.730-0.986) | 0.032 |
| m0_glszm_LargeAreaHighGrayLevelEmphasis_deciles | 1.191 | (1.062-1.336) | 0.003 |
| m0_proportion | 0.375 | (0.169-0.83) | 0.016 |
| m1_glrlm_ShortRunLowGrayLevelEmphasis_val | 0.821 | (0.694-0.970) | 0.021 |
| m1_glszm_SmallAreaHighGrayLevelEmphasis_val | 1.135 | (1.023-1.258) | 0.016 |
| m1_ngtdm_Complexity_val | 0.813 | (0.681-0.971) | 0.023 |
| m1_firstorder_Minimum_median | 2.312 | (1.496-3.576) | 0.00016 |
| m1_glszm_SmallAreaLowGrayLevelEmphasis_median | 1.218 | (1.084-1.367) | 0.00087 |
| m1_ngtdm_Busyness_median | 0.822 | (0.708-0.954) | 0.01 |
| m2_glcm_Imc2_mean | 0.866 | (0.758-0.989) | 0.033 |
| m2_gldm_LargeDependenceLowGrayLevelEmphasis_val | 1.095 | (1.003-1.197) | 0.044 |
| m2_gldm_DependenceVariance_median | 1.134 | (1.029-1.251) | 0.011 |
| m2_gldm_LargeDependenceLowGrayLevelEmphasis_median | 1.112 | (1.031-1.199) | 0.0057 |
| m2_gldm_SmallDependenceLowGrayLevelEmphasis_median | 1.154 | (1.014-1.312) | 0.029 |
| m2_glszm_LargeAreaLowGrayLevelEmphasis_deciles | 1.100 | (1.014-1.194) | 0.022 |
| m2_gldm_SmallDependenceHighGrayLevelEmphasis_deciles | 0.836 | (0.725-0.965) | 0.015 |
| m3_firstorder_Minimum_mean | 1.395 | (1.061-1.835) | 0.017 |
| m3_firstorder_Range_mean | 0.876 | (0.779-0.985) | 0.026 |
| m3_glcm_MaximumProbability_mean | 1.394 | (1.26-1.542) | 1.20E-10 |
| m3_glrlm_GrayLevelNonUniformity_mean | 1.165 | (1.037-1.309) | 0.010 |
| m3_glszm_SmallAreaLowGrayLevelEmphasis_mean | 1.138 | (1.039-1.245) | 0.0052 |
| m3_gldm_DependenceVariance_mean | 1.330 | (1.175-1.506) | 6.60E-06 |
| m3_glszm_ZoneEntropy_val | 0.813 | (0.675-0.979) | 0.029 |
| m3_gldm_DependenceVariance_val | 1.188 | (1.07-1.32) | 0.0013 |
| m3_glszm_SizeZoneNonUniformity_median | 1.149 | (1.008-1.311) | 0.038 |
| m3_firstorder_Variance_deciles | 0.847 | (0.721-0.994) | 0.042 |
| m3_glcm_InverseVariance_deciles | 1.156 | (1.031-1.297) | 0.013 |
| m3_ngtdm_Busyness_deciles | 1.182 | (1.046-1.335) | 0.0073 |
| m4_firstorder_Kurtosis_deciles | 0.843 | (0.72-0.987) | 0.033 |
| m4_gldm_DependenceNonUniformityNormalized_deciles | 0.788 | (0.664-0.935) | 0.0065 |
| m4_gldm_SmallDependenceHighGrayLevelEmphasis_deciles | 0.863 | (0.748-0.995) | 0.042 |
| m5_shape_Elongation_val | 0.883 | (0.813-0.96) | 0.0033 |
| m5_glrlm_ShortRunLowGrayLevelEmphasis_val | 0.854 | (0.735-0.993) | 0.041 |
| m5_ngtdm_Strength_val | 1.147 | (1.036-1.27) | 0.0085 |
| m5_firstorder_Minimum_median | 1.563 | (1.117-2.188) | 0.0092 |
| m5_glrlm_LongRunEmphasis_median | 1.14 | (1.038-1.252) | 0.0061 |
| m5_glrlm_ShortRunLowGrayLevelEmphasis_median | 1.179 | (1.038-1.341) | 0.012 |
| m5_glcm_Correlation_deciles | 1.183 | (1.04-1.346) | 0.011 |
| m5_ngtdm_Contrast_deciles | 1.204 | (1.062-1.365) | 0.0037 |

∗m0, normal liver tissue; m1, portal area; m2, fibrosis; m3, lymphocyte concentration area; m4, tumor region; m5, hemorrhage/necrotic area.
