# Supplementary material for: Exploring pathological signatures for predicting the recurrence of early-stage hepatocellular carcinoma based on deep learning
Source: Front Oncol. 2022 Aug 19;12:968202. doi: 10.3389/fonc.2022.968202 (PMC9439660; doi:10.3389/fonc.2022.968202)
Supplement: Supplementary file 5 [file Table_3.docx]

**Table S3. The *p* values of comparison by ROC curves for HS and CS.**

| RFS | Cohorts | |
| --- | --- | --- |
|  | Training cohort | Validation cohort |
| 1-year | 0.899 | 0.064 |
| 3-year | 0.571 | 0.618 |
| 5-year | 0.357 | 0.513 |

∗ HS, histological score; CS, combined score; RFS, recurrence-free survival.
